# The *MLH1* polymorphism rs1800734 and risk of endometrial cancer with microsatellite instability

**DOI:** 10.1186/s13148-020-00889-3

**Published:** 2020-07-08

**Authors:** Holly Russell, Katarzyna Kedzierska, Daniel D. Buchanan, Rachael Thomas, Emma Tham, Miriam Mints, Anne Keränen, Graham G. Giles, Melissa C. Southey, Roger L. Milne, Ian Tomlinson, David Church, Amanda B. Spurdle, Tracy A. O’Mara, Annabelle Lewis

**Affiliations:** 1 Cancer Gene Regulation Group, Wellcome Trust Centre for Human Genetics, University of Oxford, Roosevelt Drive, Oxford, OX3 7BN UK; 2 Cancer Genomics and Immunology Group, Wellcome Trust Centre for Human Genetics, University of Oxford, Roosevelt Drive, Oxford, OX3 7BN UK; 3 Colorectal Oncogenomics Group, Department of Clinical Pathology, The University of Melbourne, Melbourne, Victoria 3010 Australia; 4 Genomic Medicine and Family Cancer Clinic, Royal Melbourne Hospital, Parkville, Victoria 3010 Australia; 5 University of Melbourne Centre for Cancer Research, Victorian Comprehensive Cancer Centre, Parkville, Victoria 3010 Australia; 6 Department of Molecular Medicine and Surgery, Karolinska Institutet, Stockholm, Sweden; 7 Department of Clinical Genetics, Karolinska University Hospital, Stockholm, Sweden; 8 Department of Women’s and Children’s Health, Karolinska Institutet, Stockholm, Sweden; 9 Department of Laboratory Medicine, Division of Pathology, Karolinska Institutet, Karolinska University Hospital, Stockholm, Sweden; 10 Centre for Epidemiology and Biostatistics, Melbourne School of Population and Global Health, The University of Melbourne, Melbourne, Victoria 3010 Australia; 11 Cancer Epidemiology Division, Cancer Council Victoria, Melbourne, Victoria 3004 Australia; 12 Precision Medicine, School of Clinical Sciences at Monash Health, Monash University, Clayton, Victoria 3168 Australia; 13 Cancer Genetics and Evolution Laboratory, Cancer Research UK Edinburgh Centre, MRC Institute of Genetics & Molecular Medicine, The University of Edinburgh, Western General Hospital, Crewe Road South, Edinburgh, EH4 2XR UK; 14 Department of Genetics and Computational Biology, QIMR Berghofer Medical Research Institute, QLD, Brisbane, 4006 Australia; 15 Division of Biosciences, Department of Life Sciences, College of Health and Life Sciences, Brunel University, Kingston Lane, Uxbridge, UB8 3PH UK

**Keywords:** Endometrial cancer, Mismatch repair pathway, Microsatellite instability, Single nucleotide polymorphism, MLH1, rs1800734

## Abstract

Both colorectal (CRC, 15%) and endometrial cancers (EC, 30%) exhibit microsatellite instability (MSI) due to *MLH1* hypermethylation and silencing. The *MLH1* promoter polymorphism, rs1800734 is associated with MSI CRC risk, increased methylation and reduced *MLH1* expression. In EC samples, we investigated rs1800734 risk using MSI and MSS cases and controls. We found no evidence that rs1800734 or other *MLH1* SNPs were associated with the risk of MSI EC. We found the rs1800734 risk allele had no effect on *MLH1* methylation or expression in ECs. We propose that *MLH1* hypermethylation occurs by different mechanisms in CRC and EC.

## Introduction

Endometrial cancer (EC) is the most common gynaecological cancer in the developed world. Defects in the mismatch repair (MMR) pathway are common in EC, with up to 30% of tumours exhibiting loss of expression of one or more MMR proteins, high levels of microsatellite repeat instability (MSI) and hypermutation [1]. Around 15% of colorectal cancers are MSI and hypermutated [2]. These form a distinct prognostic subset with early-stage MSI CRCs having a more favourable outcome than MSS [3, 4], and MSI CRCs responding well to immunotherapy due to the abundance of neoantigens caused by hypermutation [5, 6]. Therefore, MSI status in CRC can be used both as an independent marker of CRC prognosis and a predictor of therapeutic response. In EC however, despite the prevalence of MSI, there are conflicting reports about whether or how it is associated with patient prognosis [1, 7–10]. There is also a lack of evidence for MSI as a predictive marker of therapeutic response in EC, although immunotherapies have now been approved for use in all MSI- and MMR-deficient tumours, so this data should be forthcoming [11].

MutL homologue 1 (*MLH1*) is the most commonly disrupted MMR gene in both CRC and EC. This is predominantly due to somatic silencing by promoter hypermethylation in both types of cancer, and less frequently caused by germline pathogenic variants [12]. In CRC a promoter polymorphism, rs1800734 in the 5′untranslated region of *MLH1* is strongly associated with an increased risk of MSI cancer, as well as hypermethylation and reduced *MLH1* transcription [13–17]. This polymorphism has no association with microsatellite stable (MSS) CRC and shows a much weaker association in data sets unstratified by MSI status. Using artificially de-methylated MSI CRC cell lines heterozygous for rs1800734, we have previously shown that methylation accumulation occurs more quickly on the risk (A) allele than the protective (G) allele and that this is accompanied by an allelic bias in *MLH1* transcription, with more expression from the protective allele [16]. We have suggested that the risk allele is more prone to methylation accumulation due to disruption of the binding site of transcription factor TFAP4, which binds strongly to the protective allele only [16, 18, 19].

In EC, given the prevalence of MSI cancers with *MLH1* epigenetic silencing, we also aimed to determine whether rs1800734 is also associated with the risk of MSI EC. Existing GWAS studies have not been stratified by MSI status so any MSI specific associations were unlikely to have been detected [20]. We performed a candidate association study of single nucleotide polymorphisms (SNPs) in the *MLH1* promoter region in four EC case-control sample sets stratified by MMR protein expression status. We have also investigated the effects of rs1800734 genotype on *MLH1* methylation and expression in ECs. To assess the role of rs1800734 in a dynamic system, we de-methylated an MSI EC cell line heterozygous for rs1800734 and studied allele-specific methylation accumulation and *MLH1* mRNA expression.

## Results and discussion

**Fig. 1:**
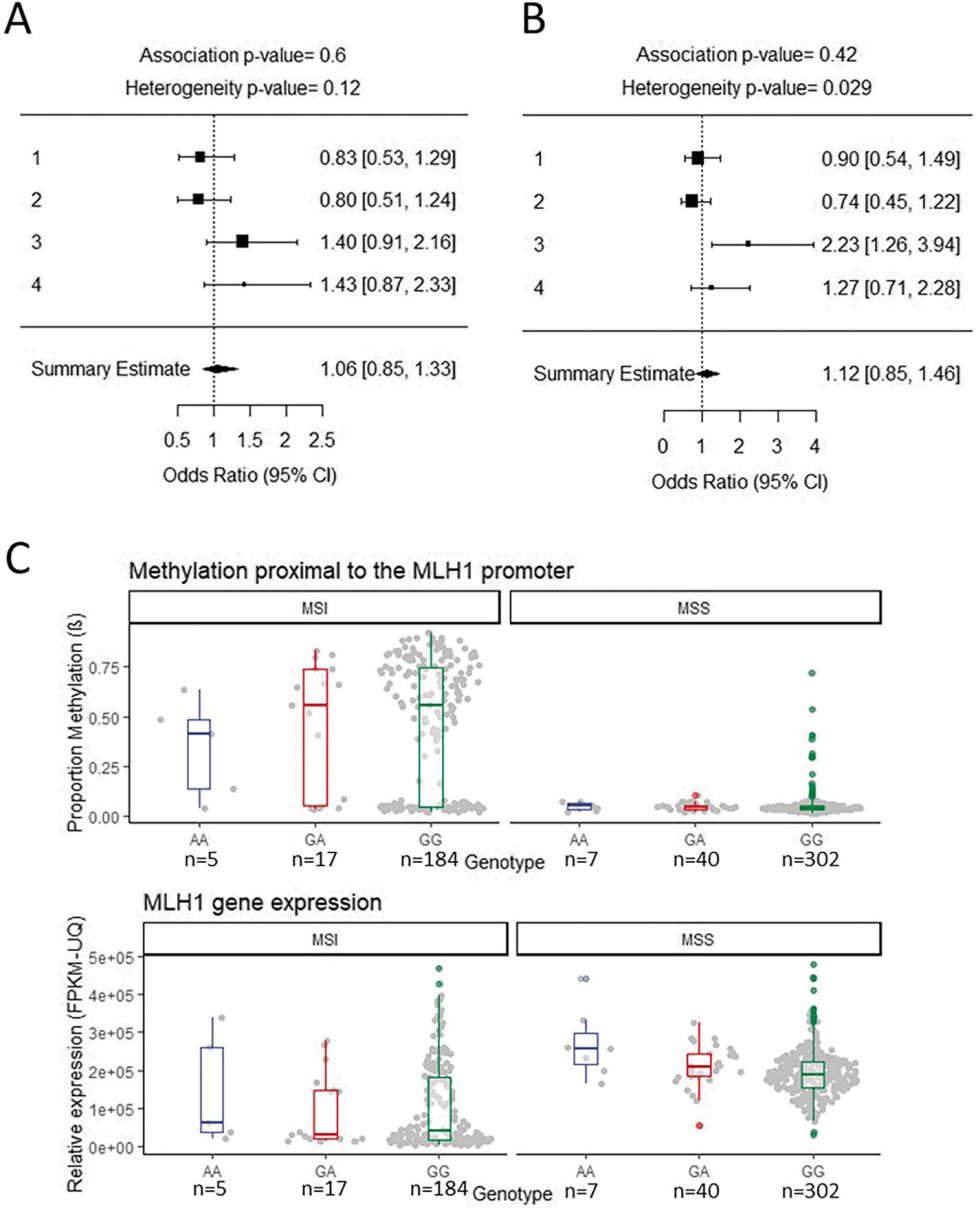
rs1800734 shows no evidence of association with endometrial cancer risk, *MLH1* promoter methylation or *MLH1* gene expression. **a** A forest plot showing a meta-analysis of four rs1800734 endometrial cancer association analyses performed on MSI cases (overall cases = 225, overall controls = 13582). The plot shows the odds ratio [upper 95% CI, lower 95% CI] of the respective studies. Diamond indicates overall odds ratio and 95% confidence interval with the *p* values generated from a fixed-effects meta-analysis showing no evidence of an association for the rs1800734 SNP with MSI endometrial cancer (*p* = 0.6). Studies included are as follows: (1) ANECS-Illumina genotyped, (2) ANECS-ICOGS genotyped, (3) RENDOCAS and (4) MCCS. **b** A forest plot showing a meta-analysis of four rs1800734 endometrial cancer association analyses performed on MSI cases which show loss of MLH1/PMS2 proteins but not those showing loss of MSH2/MSH6 (overall cases = 157, overall controls = 13582). The plot shows the odds ratio [upper 95% CI, lower 95% CI] of the respective studies. Diamond indicates overall odds ratio and 95% confidence interval with the *p* values generated from a fixed-effects meta-analysis showing no evidence of an association for the rs1800734 SNP with endometrial cancer with loss of MLH1/PMS2 (*p* = 0.42). Studies included are as follows: (1) ANECS-Illumina genotyped, (2) ANECS-ICOGS genotyped, (3) RENDOCAS and (4) MCCS. Interestingly, in the RENDOCAS study, rs1800734 does show a significant association with EC; however, this is based on 25 cases only. **c** A boxplot of the proportion of methylation proximal to the *MLH1* promoter and *MLH1* gene expression in EC patient samples (TCGA-UCEC) stratified by rs1800734 genotype and MSI/MSS status. Methylation beta (β) indicates the median proportion of methylated to unmethylated reads of 3 CpG probes proximal to *MLH1* (probe names: cg00893636, cg02279071, cg13846866). Relative expression (FPKM-UQ) indicates fragments per kilobase of *MLH1* per million mapped reads upper quartile. Plots show the median, upper and lower quartile of expression or methylation stratified by MSI status and rs1800734 genotype (MSI *n* = 206, MSS *n* = 349). rs1800734 genotype had no significant effect on methylation (*p* = 0.556 MSS, *p* = 0.585 MSI; Kruskal-Wallis) or expression (*p* = 0.434 MSI, Kruskal-Wallis) except for a small set of MSS ECs with AA genotype (*n* = 7) in which expression was significantly higher than MSS ECs with the GG genotype (*p* = 0.045, pairwise Wilcoxon). The biological significance of this is uncertain

We inferred MSI status, using MMR protein expression levels, on patients from four endometrial cancer datasets previously used for published genome-wide association studies [20, 21]. We then carried out association analyses for rs1800734 and 126 other SNPs in a 1 Mb region centred on the *MLH1* transcriptional start site on all MSI and MSS cases vs controls for each study (total numbers used in the meta-analysis were the following: MSI *n* = 225, MSS *n* = 563, controls *n* = 13,582, consisting of ANECS-Illumina genotyped, ANECS-iCOGS genotyped, RENDOCAS, MCCS, Fig. 1a; detailed numbers are broken down in supplementary table 1). We assessed all the SNPs in the *MLH1* promoter and surrounding regions to cover all SNPs in LD with rs1800734 (only 3 SNPs with *r*^2^ > 0.5) and allow for the possibility that variants in binding sites of transcription factors (TFs) other than TFAP4 are more important for regulating *MLH1* transcription in endometrial cell types. SNPs within in silico*-*predicted TF binding sites, and any known functional role of these TFs in EC, are shown in supplementary table 2. We carried out a meta-analysis and found no evidence of MSI EC risk association for rs1800734 (OR = 1.06 CI 0.85–1.33 *p* = 0.60) or any other SNPs in the *MLH1* region (supplementary table 2), after correction for multiple testing. While the sample set is relatively small and the findings will need replicating, a similarly sized MSI CRC sample set gave a strong rs1800734 risk association (CRC MSI cases *n* = 170, controls *n* = 2686, OR = 1.95, 95% CI 1.50–2.55, *p* = 8.04 × 10^−7^, [16]). We estimate that we had 99% power to detect an OR of this magnitude for MSI EC. However, unlike CRCs where the majority of sporadic MSI tumours occur as a result of *MLH1* silencing due to promoter hypermethylation [22, 23], a significant proportion of MSI EC occurs as a result of loss of MSH2/MSH6 protein expression as opposed to MLH1/PMS2 (24% 41/173 from ANECS-Illumina, ANECS-iCOGS, MCCS). We hypothesized that the difference in proportion of MLH1 expressing samples could explain some of the difference in rs1800734 risk association between CRC and EC. We therefore carried out a further meta-analysis (Fig. 1b) selecting only EC samples with loss of MLH1/PMS2 protein expression and omitting those in which the MSI was accompanied by loss of MSH2/MSH6 or where MMR protein expression data was not available. This focussed meta-analysis also found no evidence of an association between rs1800734 and MLH1/PMS2 deficient EC risk (MLH1 loss cases *n* = 157, controls *n* = 13,582, OR = 1.12, CI 0.85–1.46 *p* = 0.42, supplementary table 3, power calculations as above indicate a power of 95% with this smaller sample size)

In CRC tumour tissue stratified by genotype (but not in normal tissue), we previously found that rs1800734 acted as an *MLH1* expression quantitative trait locus (eQTL) and a methylation quantitative trait locus meQTL (meQTL), with the risk allele associated with higher methylation and lower mRNA expression [16]. We were therefore interested to see whether this was true for EC tumours given that there was no association observed between rs1800734 and EC risk. Using publicly available data from the Cancer Genome Atlas (TCGA-UCEC) we assessed the association of rs1800734 status on *MLH1* promoter methylation and *MLH1* mRNA expression, stratified by MSI status (TCGA-UCEC MSI *n* = 206; MSS *n* = 349; Fig. 1c). No significant differences were found between rs1800734 genotypes for either methylation or mRNA expression. The MSI samples were further classified into high-instability levels (MSI-h) and low (MSI-l) but neither of these subsets showed any significant differences in methylation or expression by genotype (supplementary table 4). Due to lack of complete data on MMR protein expression in TCGA-UCEC samples, we used *MLH1* promoter median methylation levels (median beta > 0.2) in MSI positive samples to infer MSI caused specifically by MLH1 loss. In this subset, no significant differences were found between rs1800734 genotypes for methylation or mRNA expression (TCGA-UCEC MSI high methylation *n* = 135; MSS *n* = 349; supplementary figure 1). This was despite a significant negative correlation between *MLH1* methylation and mRNA expression levels (Pearson coefficient = − 0.86, *p* = 2.2 × 10^−16^; supplementary figure 2), supporting the prevailing theory that methylation is the primary mechanism of *MLH1* silencing in EC.

**Fig. 2:**
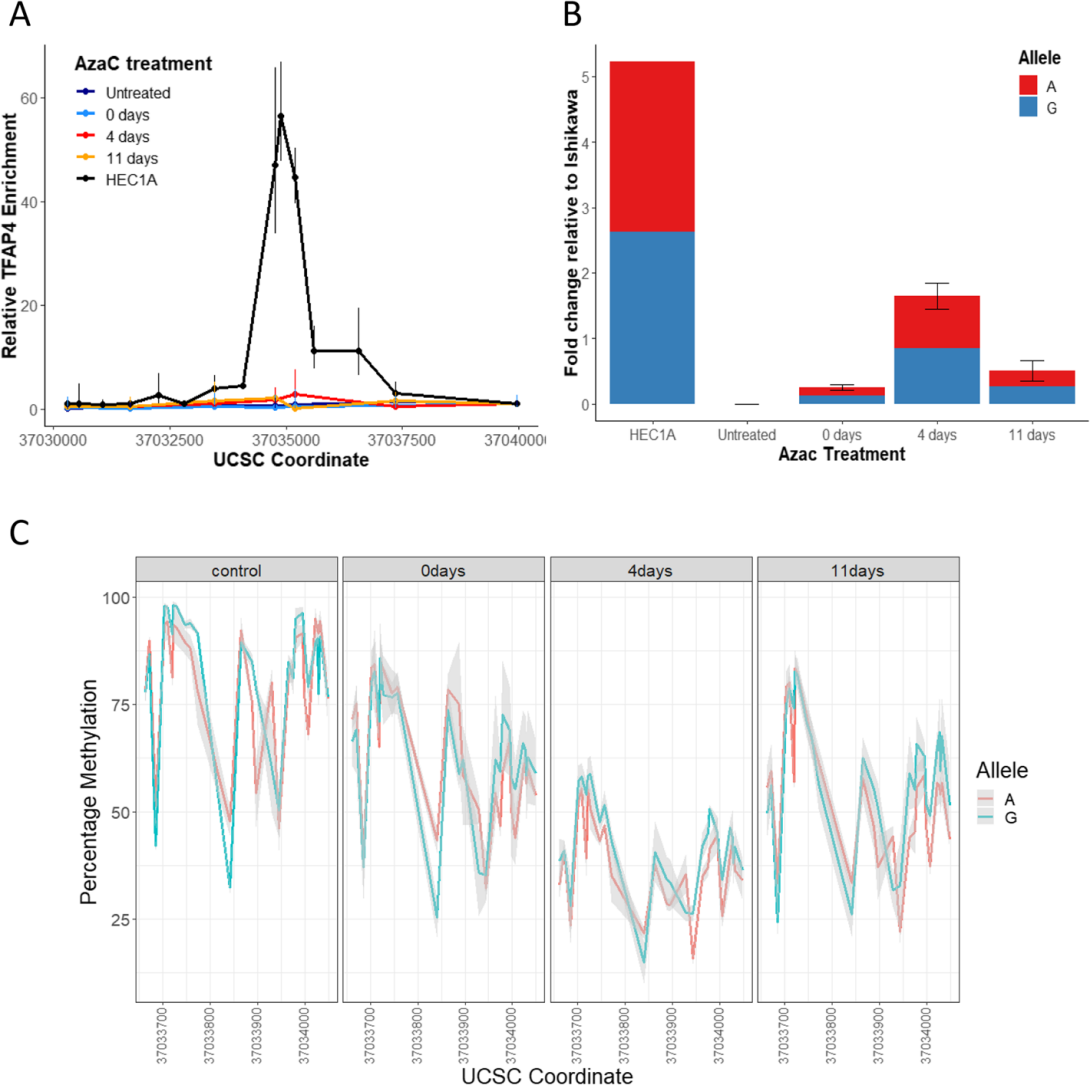
TFAP4 binding occurs in EC cells but does not result in rs1800734 allelic bias of *MLH1* expression or promoter methylation. **a** TFAP1 binds to MSS but not MLH1 methylated MSI EC cells. The line graph shows relative TFAP4 enrichment at UCSC coordinates proximal to the *MLH1* promoter in HEC1A (MSS) cells and NOU1 (MSI) cells untreated and 0 days, 4 days and 11 days post 48 h 5′-azacytidine. Relative TFAP4 enrichment was determined after normalization with input DNA using the ΔΔCT method. Error bars show the standard error of the mean (*n* = 3). HEC1A cells show TFAP4 binding but AzaC treatment did not reactivate TFAP4 expression in Nou1 cells. **b***MLH1* expression in EC cells shows no rs1800734 allelic bias. RNA was extracted from HEC1A cells and NOU1 cells, untreated, 0 days, 4 days and 11 days post 48 h 5′-azacytidine treatment. The bar chart shows relative mRNA expression levels with error bars showing the standard error of the mean (*n* = 3). Percentages represent the proportion of G or A reads out of the total rs1800734 sequences for each cell line/time point. NOU1 *MLH1* expression is activated by treatment with 5-azacytidine, with the highest expression 0 days post-treatment. Re-repression occurs at 4 days and 11 days post-treatment. There is no significant allelic bias at any stage. **c** 5′-Azacytidine treatment of NOU1 cells removes *MLH1* promoter methylation but no allelic bias is seen in the control or at any post-treatment time point as methylation is re-acquired

We hypothesized that TFAP4 may not be present in endometrial tissues and could therefore offer no protection against promoter methylation accumulation on the protective rs1800734 allele. However, data from the GTEx portal showed expression of TFAP4 in uterine tissue at equivalent or greater levels than intestinal tissues (Common Fund (CF) Genotype-Tissue Expression Project (GTEx) dbGaP Study Accession, phs000424.v8.p2). We therefore checked the activity of the specific TFAP4 binding site at rs1800734 in endometrial cells by carrying out chromatin immunoprecipitation in EC cell lines: one MSI cell line (NOU1, with *MLH1* promoter methylation) and one MSS cell line (HEC1A, Fig. 2a). TFAP4 did bind at or near rs1800734 in the MSS line and, interestingly, there was a strong allelic bias in its binding as we and others previously observed in CRC cells (supplementary figure 3). As expected, no binding was detected in MSI cells so we treated with 5 Aza-Cytosine to remove methylation and then measured *MLH1* methylation, expression and TFAP4 binding. Our findings differed substantially from CRC cell lines. TFAP4 binding was only restored at very low levels (Fig. 2a). Although some *MLH1* re-expression occurred (Fig. 2b) and promoter methylation was removed and re-established (Fig. 2c), there was no significant allelic bias in either expression or methylation at any stage.

In this small candidate study, our results suggest that SNP rs1800734 in the *MLH1* promoter may not be associated with risk of MSI EC, even though this SNP shows a strong association with MSI CRC. This is despite EC and CRC sharing a common mechanism of epigenetic silencing of *MLH1* transcription. The SNP acts as a meQTL and eQTL in CRC but not in EC. In addition, it has allele-specific effects on methylation accumulation and mRNA expression levels in dynamic CRC cell line systems but not in EC cells. Since both cell types exhibit allele-specific TFAP4 binding, we conclude that this is not sufficient for the establishment of meQTL and eQTLs.

Previous findings by Fang et al. [24] have implicated the transcriptional repressor MAFG and cofactors including the de novo methylase DNMT3B in the accumulation of methylation at *MLH1* in CRC. MAFG becomes stabilized by phosphorylation in *BRAF*V600E mutated cells leading to hypermethylation at several promoters, including *MLH1,* and a CpG island methylator phenotype (CIMP). Interestingly, *BRAF*V600E mutations occur commonly in somatic MSI CRC but are very rarely found in MSI EC [25, 26]. Our MSI CRC cell line model (CO-115) but not our MSI EC model (NOU1) carried a *BRAF*V600E mutation. This observation could explain some or all of the differences we see in rs1800734 and cancer risk association between CRC and EC and the lack of any genotype-specific *MLH1* methylation and expression bias in EC tumours and cell lines.

**Fig. 3:**
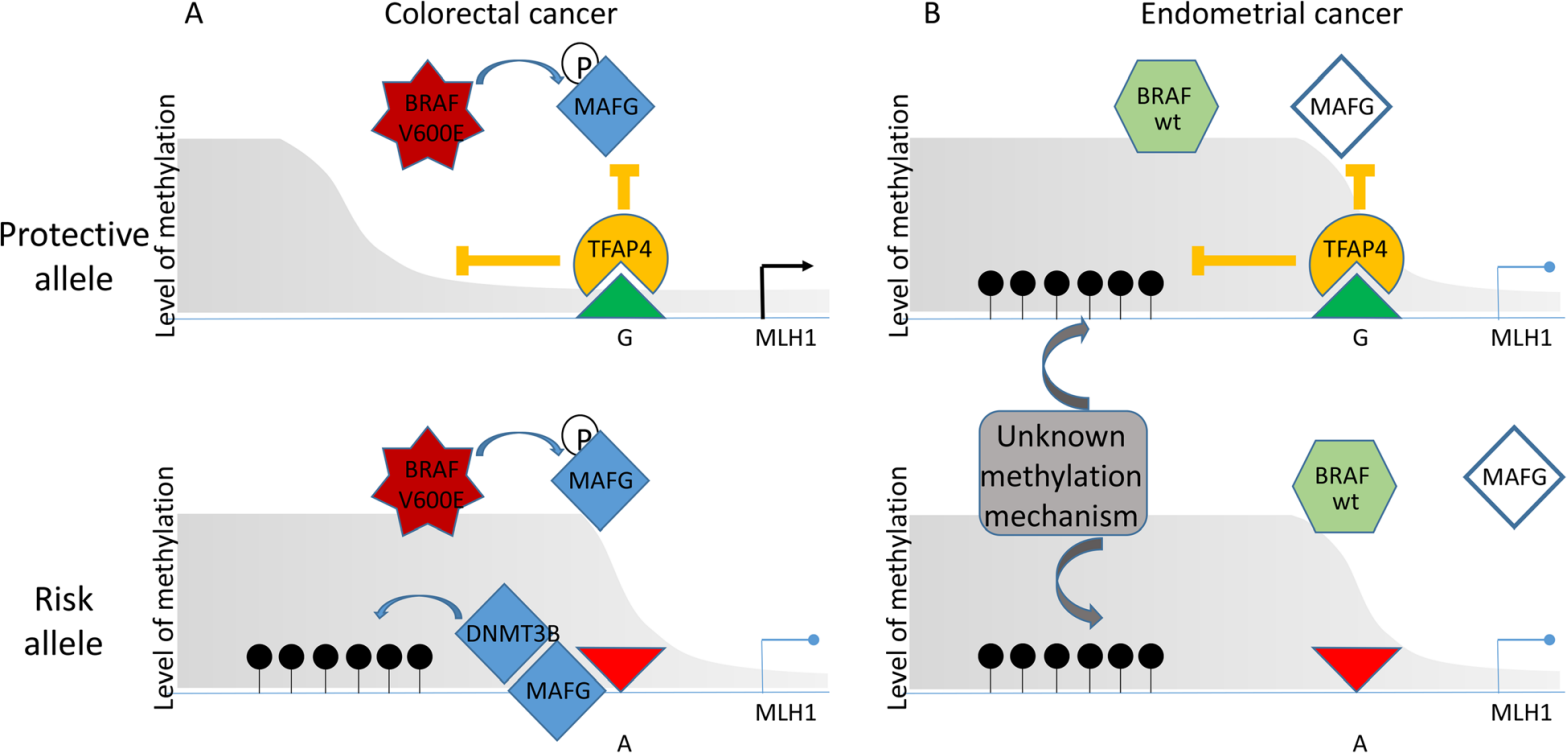
Oncogenic BRAFV600E mutation in colorectal cancer allows *MLH1* methylation after TFAP4 disruption. **a** Colorectal cancer: A BRAFV600E mutation activates the MEK/ERK pathway to phosphorylate MAFG, allowing DNMT3B recruitment. TFAP4 binding sterically hinders MAFG on the protective (G) rs1800734 allele, preventing DNMT3B recruitment and subsequent *MLH1* methylation. TFAP4 binding is disrupted on the rs1800734 risk (A) allele leading to DNMT3B mediated *MLH1* promoter methylation and transcriptional repression. **b** Endometrial cancer: *BRAF* is rarely mutated and *MLH1* methylation does not occur via the MAFG pathway. TFAP4 is still able to bind the protective allele but this has no significant effect on methylation accumulation

Figure 3 outlines the proposed mechanism to explain the difference in the role of rs1800734 in CRC and EC. In the presence of mutant BRAF, TFAP4 and/or cofactor binding on the protective allele of the SNP reduces methylation accumulation via MAFG and DNMT3. However, in EC when no BRAF mutations are present, the methylation is acquired via a different unknown mechanism which is unaffected by TFAP4 binding. Other transcription factors with binding sites upstream of *MLH1*, particularly those known to be associated with EC, merit further investigation.

The pathways to cancer and combination of driver mutations in EC are poorly understood in comparison with CRC. No EC driver mutations associated with hypermethylation of promoters are currently known so an important next step is to uncover the key mutations responsible for *MLH1* methylation and CIMP initiation in EC.

## Methods

### MMR assessment

Tumour MMR expression data was previously generated by immunohistochemistry (IHC) and assessed as described (ANECS [27], RENDOCAS [28], MCCS [29, 30]). Briefly, cases with nuclear staining of all MMR proteins in tumour cells were considered MMR-proficient and classified as MSS. Cases were reported as MMR-deficient when tumour cells showed total or partial nuclear loss of expression in one or more of the MMR proteins and were classified as MSI.

### Candidate SNP meta-analysis

GWAS data for meta-analysis was collated from four endometrial cancer genome-wide association studies [20, 21, 31]—Australian National Endometrial Cancer Studies (ANECS-Illumina genotyped, ANECS-ICOGS genotyped), Registry of Endometrial Cancer in Sweden (RENDOCAS) and Melbourne Collaborative Cohort Study (MCCS). IMPUTE2 was used to impute genotypes to the positive strand of the 1000 Genomes project, v3, phase 1 dataset. Cases were of European ancestry with a confirmed EC diagnosis. Genotyping in each study was performed as previously described [20, 21]: ANECS-Illumina (MSI *n* = 66, MSS *n* = 254, controls *n* = 3,083) with Illumina Infinium 610K; ANECS-iCOGS (MSI *n* = 67, MSS *n* = 156, controls *n* = 1,956) and RENDOCAS (MSI *n* = 52, MSS *n* = 88, controls *n* = 7563) with an Illumina custom array designed by the Collaborative Oncological Gene environment Study initiative (iCOGS) [20] and MCCS (MSI n = 40, MSS n = 65, controls n = 980) with the Illumina OncoArray 534K genotyping ChIP [21]. Controls were country-matched to cases and genotyped using the same platforms*.*

Total numbers used in the meta-analysis were as follows: MSI *n* = 225, MSS *n* = 563 and controls *n* = 13,582. Quality control consisted of exclusion of SNPs with < 95% call rates, MAFs < 1%, duplicated results or related individuals. Comprehensive sequencing for germline mutations has not been completed for all ANECS and RENDOCAS studies so it is possible a small number (< 3%) of undiagnosed Lynch syndrome patients are present in the data. SNPs for this candidate study were limited to those within chromosome 3, 1Mb upstream and downstream of *MLH1* transcriptional start site (chr3:36,000,000–38,000,000 hg38; chr3:36024996–38024996 hg19). rs1800734 was directly genotyped in all datasets. To determine if our dataset (MSI and controls) was of a sufficient size, power calculations based on our CRC association study (OR = 1.95, MAF of 0.2, *n* = 13807, case rate = 0.016) indicated a power of 99% to discover a similar association to that seen in CRC. Using a more conservative OR of 1.4 in the same calculation indicated a power of 85%. Association statistics from individual GWAS’s were entered into PLINK 1.9 for a fixed-effects meta-analysis. *P*-threshold for candidate significance was 0.05. Standard Bonferroni methods were used to correct *P*-threshold for multiple testing. Confidence intervals are set at 95%.

### TCGA-UCEC analysis

TCGA-UCEC methylation, gene expression data and MSI status were downloaded from the GDC portal (https://portal.gdc.cancer.gov/) using the GDC toolkit. The rs1800734 genotype was extracted from TCGA-UCEC whole genome sequencing sliced BAM files using Platypus variant calling software [24]. Data was downloaded, collated and pre-analysed using a custom script available on GitHub (https://github.com/kzkedzierska/mlh1_endo). For *MLH1* promoter methylation, the beta median methylation level for CpG residues proximal (± 2000 bp) to rs1800734 was calculated. *MLH1* transcript fragments per kilobase per million mapped reads upper quartile (FPKM-UQ) was used as a measure of expression. Samples with any missing values were excluded before data visualization and statistical analysis in R (MSI *n* = 206; MSS *n* = 349).

### Cell lines

HEC1A and NOU1 cells were maintained in Dulbecco’s modified eagle medium (Gibco™), 10% FBS, 0.1% penicillin-streptomycin. rs1800734 was genotyped using KASPAR^TM^ technology (LGC) according to the manufacturer’s instructions using specific primers (Supplementary table 5).

### Analysis of methylation

DNA was extracted from fresh cells using the DNeasy kit (QIAGEN). Bisulphite conversion of DNA was carried out using the EZ DNA methylation kit (Zymo Research) according to the manufacturer’s instructions. Converted DNA was amplified with Pyromark PCR kit (Qiagen) using CpG free primers (Supplementary table 5) with Illumina-specific sequence tags to ensure unbiased amplification of methylated and unmethylated template. Amplicons from each sample were barcoded together using a custom set of index tags and primers [32]. Sequencing was carried out using a 250-bp paired end kit on a MiSeq (Illumina) according to the manufacturer’s instructions. MiSeq output was demultiplexed and FASTQ files generated (Basespace, Illumina). The sequences were quality assessed and trimmed (FastQC and TrimGalore, Babraham Bioinformatics) then aligned and the methylation called by rs1800734 allele (Bismark, Babraham Bioinformatics).

### Analysis of mRNA

RNA was extracted from fresh cells using the RNeasy kit (QIAGEN) and cDNA was generated (High Capacity cDNA Reverse Transcription Kit, Applied Biosystems) according to the manufacturer’s instructions. Gene expression was quantified and normalized using Taqman gene expression ready mixed assays (Applied Biosystems, Thermofisher). Allele-specific *MLH1* expression was assessed by amplification of cDNA using Illumina tagged primers (Supplementary table 5) followed by NGS sequencing on a MiSeq (Illumina) as above. Trimmed FastQ sequences were aligned using bwa-mem and the rs1800734 variant called by Platypus [33].

### Chromatin Immunoprecipitation

Approximately 10^8^ cells were crosslinked for 10 min with 1% formaldehyde, neutralized with 125 mM glycine, washed with ice-cold PBS and scraped. After 2 further PBS washes, cells were resuspended in lysis buffer, (1% SDS, 10 mM EDTA, 50 mM Tris-HCl, protease inhibitors) sonicated using a Bioruptor (Diagenode) for 7-15 x 15 s cycles, centrifuged at max speed for 10 min at 4 °C and diluted 1:10 in IP dilution buffer (1% triton-100, 2 mM EDTA, 150 mM NaCl, 20 mM Tris). Immunoprecipitation (IP) with 5 μg of antibody (anti-TFAP4 Santa Cruz Biotechnology, sc-18593X) was carried out overnight at 4 °C and then incubated for 4 h with 50 μl of protein G Dynabeads (Invitrogen). For each chromatin sample, a mock IP with no antibody was carried out in parallel with the TFAP4 IP, and for all subsequent steps of the assay, as a negative control. Bead/antibody and mock complexes were washed with TSEI (0.1% SDS, 1% TritonX-100, 2 mM EDTA, 20 mM Tris, 150 mM NaCl), TSEII (0.1% SDS, 1% TritonX-100, 2 mM EDTA, 20 mM Tris, 500 mM NaCl), LiCl buffer (0.25LiCl, 1% NP-40, 1% deoxycholate, 1 mM EDTA, 10 mM Tris-HCl) and TE according to standard protocols and eluted with 1% SDS, 0.1 M NaHCO_3_. One microliter of DNA was analysed in duplicate or triplicate by SYBR green qPCR using PowerUp SYBR™ Green Master Mix (Thermofisher) and primers covering the *MLH1* promoter region (Supplementary Table 6). The results were calculated with the ∆∆CT method using Ct values from the input chromatin to normalize (*∆*CT) and then expressed relative to a primer set outside the TFAP4 binding site (∆∆Ct) and the relative fold change calculated using the equation 2^−∆∆Ct^. No amplification was observed from DNA extracted from the mock IPs.

### 5-Aza-2′-deoxycytidine treatment

Adherent semiconfluent MSI NOU1 cells in exponential growth were treated with 5uM 5-Aza-2′-deoxycytidine in standard medium (AzaC, Sigma A3656) for 48 h (with replenishment of AzaC after 24 h). AzaC was removed and cells washed with PBS and then cultured in standard medium for 0, 4, 7 and 11 days. RNA and DNA were extracted simultaneously using the AllPrep kit (Qiagen) and *MLH1* mRNA expression and promoter methylation assessed as described above. ChIP was carried out post AzaC treatment as described above.

### Plots and statistics

R software and associated packages (tidyverse, gridExtra, ggplot2, ggsci, dylpr and ggforce) were used to generate all graphs and carry out statistical tests including ANOVA, Tukey, Kruskal-Wallis, paired Wilcoxon, *t* test and Pearson’s. Power calculations were carried out using the genpwr package

## Supplementary information

**Additional file 1: Supplementary figure 1.** A Boxplot of the proportion of methylation proximal to the MLH1 promoter and MLH1 gene expression in EC patient samples (TCGA-UCEC) stratified by rs1800734 genotype and MSI with high MLH1 methylation (median Beta >0.2) and MSS status. Supplementary figure 2: MLH1 expression of MSI samples inversely correlates with promoter methylation. Supplementary figure 3: In HEC1A cells TFAP4 binds preferentially to the rs1800734 G allele. Supplementary table 1: Sample numbers and minor allele frequency for rs1800734 for each data set. Supplementary table 4: Statistical test p-values on genotype vs methylation and genotype vs expression associations in TCGA-UEAC sample subsets. Supplementary table 5: Primers used for genotyping, amplicon bisulphite sequencing and cDNA amplification. Supplementary table 6: Primers used for Q-PCR (SYBR) amplification of ChIP DNA.

**Additional file 2: Supplementary figure 2.** Results of meta-analysis of all MSI cases versus controls (from ANECS – Illumina genotyped, ANECS – iCOGS genotyped, RENDOCAS, MCCS; MSI *n* = 225, controls *n* = 13,582) showing association statistics for all SNPs in the MLH1 promoter region (chr3:36,000,000-38,000,000 hg38; chr3:36024996-38024996 hg19) and in silico predicted transcription factor binding sites (SNP2TFBS).

**Additional file 3: Supplementary figure 3.** Results of meta-analysis of cases with loss of MLH1/PMS2 protein expression only versus controls (from ANECS – Illumina genotyped, ANECS – iCOGS genotyped, RENDOCAS, MCCS; MLH1 loss cases *n* = 157, controls *n* = 13,582) showing association statistics for all SNPs in the MLH1 promoter region (chr3:36,000,000-38,000,000 hg38; chr3:36024996-38024996 hg19).

## Data Availability

The datasets used and analysed during the current study are available from the corresponding author on reasonable request.

## Abbreviations

ANECS: Australian National Endometrial Cancer Studies
CIMP: CpG island methylator phenotype
CRC: Colorectal cancer
EC: Endometrial cancer
eQTL: Expression quantitative trait locus
GTEx: Genotype-tissue expression project
MAF: Minor allele frequency
MCCS: Melbourne Collaborative Cohort Study
meQTL: Methylation quantitative trait locus
MLH1: MutL homologue 1
MMR: Mismatch repair
MSH2: MutS homologue 2
MSH6: MutS homologue 6
MSI: Microsatellite instability
MSS: Microsatellite stable
PMS2: PMS1 homologue 2
RENDOCAS: Registry of Endometrial Cancer in Sweden
SNP: Single nucleotide polymorphism
TCGA: The cancer genome atlas
TFAP4: Transcription factor AP4
