# Correction to: The MLH1 polymorphism rs1800734 and risk of endometrial cancer with microsatellite instability

**DOI:** 10.1186/s13148-021-01058-w

**Published:** 2021-04-01

**Authors:** Holly Russell, Katarzyna Kedzierska, Daniel D. Buchanan, Rachael Thomas, Emma Tham, Miriam Mints, Anne Keränen, Graham G. Giles, Melissa C. Southey, Roger L. Milne, Ian Tomlinson, David Church, Amanda B. Spurdle, Tracy A. O’Mara, Annabelle Lewis

**Affiliations:** 1 Cancer Gene Regulation Group, Wellcome Trust Centre for Human Genetics, University of Oxford, Roosevelt Drive, Oxford, OX3 7BN UK; 2 Cancer Genomics and Immunology Group, Wellcome Trust Centre for Human Genetics, University of Oxford, Roosevelt Drive, Oxford, OX3 7BN UK; 3 Colorectal Oncogenomics Group, Department of Clinical Pathology, The University of Melbourne, Melbourne, VIC 3010 Australia; 4 Genomic Medicine and Family Cancer Clinic, Royal Melbourne Hospital, Parkville, VIC 3010 Australia; 5 University of Melbourne Centre for Cancer Research, Victorian Comprehensive Cancer Centre, Parkville, VIC 3010 Australia; 6 Department of Molecular Medicine and Surgery, Karolinska Institutet, Stockholm, Sweden; 7 Department of Clinical Genetics, Karolinska University Hospital, Stockholm, Sweden; 8 Department of Women’s and Children’s Health, Karolinska Institutet, Stockholm, Sweden; 9 Department of Laboratory Medicine, Division of Pathology, Karolinska Institutet, Karolinska University Hospital, Stockholm, Sweden; 10 Centre for Epidemiology and Biostatistics, Melbourne School of Population and Global Health, The University of Melbourne, Melbourne, VIC 3010 Australia; 11 Cancer Epidemiology Division, Cancer Council Victoria, Melbourne, VIC 3004 Australia; 12 Precision Medicine, School of Clinical Sciences At Monash Health, Monash University, Clayton, VIC 3168 Australia; 13 Cancer Genetics and Evolution Laboratory, Cancer Research UK Edinburgh Centre, MRC Institute of Genetics & Molecular Medicine, The University of Edinburgh, Western General Hospital, Crewe Road South, Edinburgh, EH4 2XR UK; 14 Department of Genetics and Computational Biology, QIMR Berghofer Medical Research Institute, Brisbane, QLD 4006 Australia; 15 Division of Biosciences, Department of Life Sciences, College of Health and Life Sciences, Brunel University, Kingston Lane, Uxbridge, UB8 3PH UK

## Correction to: Clinical Epigenetics (2020) 12:102 https://doi.org/10.1186/s13148-020-00889-3

Following publication of the original article [1], an error was identified in the Acknowledgements section. The statement: “The research was funded/supported by the National Institute for Health Research (NIHR) Oxford Biomedical Research Centre (BRC)” should be added.

## References

[1] Russell (2020). Clin Epigenetics.

