# Supplementary material for: The MLH1 polymorphism rs1800734 and risk of endometrial cancer with microsatellite instability
Source: Clin Epigenetics. 2020 Jul 8;12:102. doi: 10.1186/s13148-020-00889-3 (PMC7346630; doi:10.1186/s13148-020-00889-3)
Supplement: Supplementary file 1 — Additional file 1: Supplementary figure 1. A Boxplot of the proportion of methylation proximal to the MLH1 promoter and MLH1 gene expression in EC patient samples (TCGA-UCEC) stratified by rs1800734 genotype and MSI with high MLH1 methylation (median Beta >0.2) and MSS status. Supplementary figure 2: MLH1 expression of MSI samples inversely correlates with promoter methylation. Supplementary figure 3: In HEC1A cells TFAP4 binds preferentially to the rs1800734 G allele. Supplementary table 1: Sample numbers and minor allele frequency for rs1800734 for each data set. Supplementary table 4: Statistical test p-values on genotype vs methylation and genotype vs expression associations in TCGA-UEAC sample subsets. Supplementary table 5: Primers used for genotyping, amplicon bisulphite sequencing and cDNA amplification. Supplementary table 6: Primers used for Q-PCR (SYBR) amplification of ChIP DNA. [file 13148_2020_889_MOESM1_ESM.docx]

Supplementary figure 1


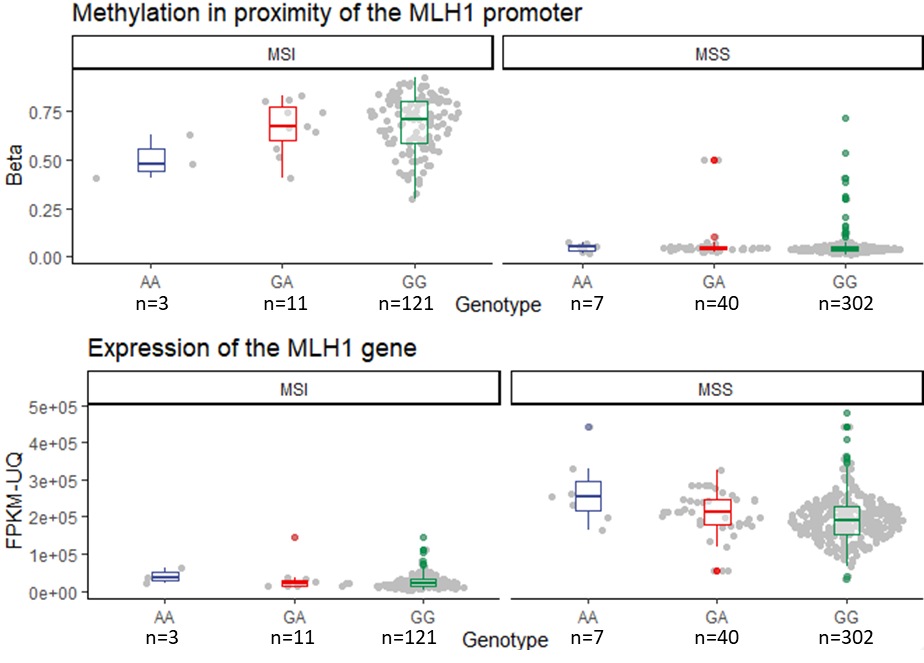


A Boxplot of the proportion of methylation proximal to the *MLH1* promoter and *MLH1* gene expression in EC patient samples (TCGA-UCEC) stratified by rs1800734 genotype and MSI with high MLH1 methylation (median Beta >0.2) and MSS status. Methylation Beta (β) indicates median proportion methylated to unmethylated reads of 3 CpG probes proximal to *MLH1* (cg00893636, cg02279071, cg13846866 probes). Relative expression (FPKM-UQ) indicates Fragments Per Kilobase of *MLH1* per Million mapped reads upper quartile. Plots show the median, upper and lower quartile of expression/methylation stratified by MSI and MLH1 methylation status and rs1800734 genotype (MSI n = 135 , MSS n = 349). rs1800734 genotype had no significant effect on methylation (p = 0.556 MSS, p= 0.973MSI; Kruskal Wallis) or expression (p= 0.079 MSI, KRUSKAL Wallis) except for a small set of MSS ECs with AA genotype (n=7) in which expression was significantly higher than MSS ECs with the GG genotype (p = 0.045, Pairwise Wilcoxon Test). The biological significance of this is uncertain.

Supplementary figure 2


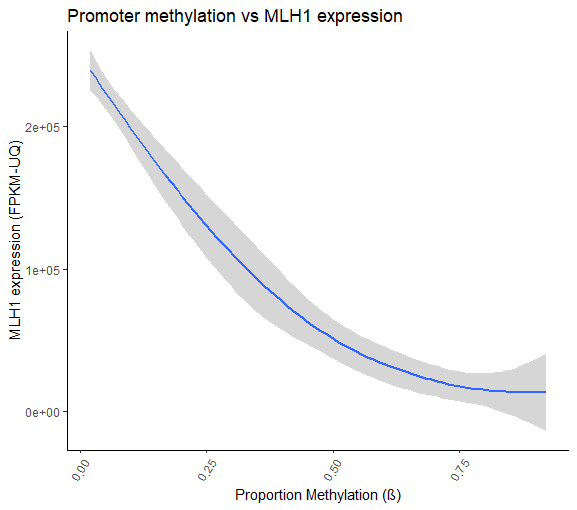


**MLH1 expression of MSI samples inversely correlates with promoter methylation.** The graph shows MSI endometrial cancer patient data extracted from TCGA (n = 206) with the proportion of promoter methylation (Median beta (β) methylation of CpG residues +/-2000 of rs1800734) plotted against MLH1 expression (FPKM-UQ). The shading represents standard error of the mean. Methylation and expression show a significant correlation (Pearson’s correlation coefficient = -0.860; p = 2.2x10^-16^).

Supplementary figure 3

*


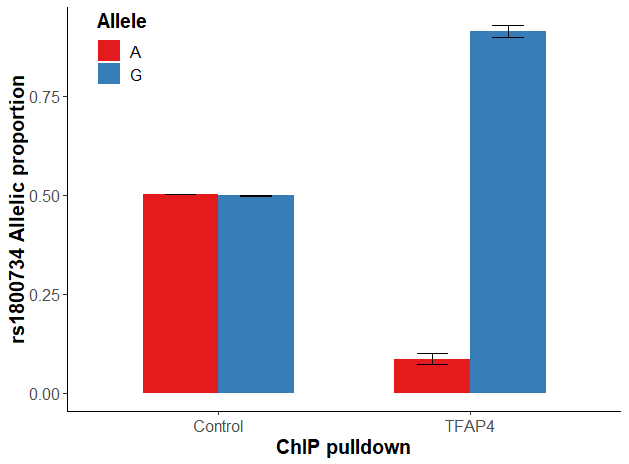


**In HEC1A cells TFAP4 binds preferentially to the rs1800734 G allele.** The graph shows the DNA pulldown from TFAP4-specific Chromatin Immunoprecipitation (ChIP) and input DNA stratified by rs1800734 allele. HEC1A DNA bound to TFAP4 was sequenced, genotyped and quantified using Illumina^TM^ technology and Platypus variant calling. Error bars represent standard error of the mean of replicates (n=2). * indicates P value < 0.001 from an independent T-test, performed by comparing the mean allelic ratio of TFAP4 pulldown vs ChIP input control.

Supplementary table 1: **Sample numbers and minor allele frequency for rs1800734 for each data set**

|  | All Cases | MSI | MLH1loss | MSS | Controls | MAF in MLH1 loss cases | MAF in Controls |
| --- | --- | --- | --- | --- | --- | --- | --- |
| ANECS-Illumina | 369 | 66 | 49 | 254 | 3083 | 0.1979 | 0.2153 |
| ANECS-ICOGS | 277 | 67 | 54 | 156 | 1956 | 0.1759 | 0.2234 |
| RENDOCAS-ICOGS | 165 | 52 | 25 | 88 | 7563 | 0.38 | 0.216 |
| MCCS - OncoArray | 134 | 40 | 29 | 65 | 980 | 0.2759 | 0.2311 |
| **Total** | **945** | **225** | **157** | **563** | **13582** |  |  |

Supplementary table 4: **Statistical test p-values on genotype vs methylation and genotype vs expression associations in TCGA-UEAC sample subsets**

Supplementary table 5: **Primers used for genotyping, amplicon bisulphite sequencing and cDNA amplification**

Supplementary table 6: **Primers used for Q-PCR (SYBR) amplification of ChIP DNA**
